# nf-core/isoseq: simple gene and isoform annotation with PacBio Iso-Seq long-read sequencing

**DOI:** 10.1093/bioinformatics/btad150

**Published:** 2023-03-24

**Authors:** Sébastien Guizard, Katarzyna Miedzinska, Jacqueline Smith, Jonathan Smith, Richard I Kuo, Megan Davey, Alan Archibald, Mick Watson

**Affiliations:** The Roslin Institute and R(D)SVS, University of Edinburgh, Edinburgh EH25 9RG, United Kingdom; The Roslin Institute and R(D)SVS, University of Edinburgh, Edinburgh EH25 9RG, United Kingdom; The Roslin Institute and R(D)SVS, University of Edinburgh, Edinburgh EH25 9RG, United Kingdom; The Roslin Institute and R(D)SVS, University of Edinburgh, Edinburgh EH25 9RG, United Kingdom; Wobble Genomics, University of Edinburgh, Edinburgh EH25 9RG, United Kingdom; The Roslin Institute and R(D)SVS, University of Edinburgh, Edinburgh EH25 9RG, United Kingdom; The Roslin Institute and R(D)SVS, University of Edinburgh, Edinburgh EH25 9RG, United Kingdom; The Roslin Institute and R(D)SVS, University of Edinburgh, Edinburgh EH25 9RG, United Kingdom

## Abstract

**Motivation:**

Iso-Seq RNA long-read sequencing enables the identification of full-length transcripts and isoforms, removing the need for complex analysis such as transcriptome assembly. However, the raw sequencing data need to be processed in a series of steps before annotation is complete. Here, we present nf-core/isoseq, a pipeline for automatic read processing and genome annotation. Following nf-core guidelines, the pipeline has few dependencies and can be run on any of platforms.

**Availability and implementation:**

The pipeline is freely available online on the nf-core website (https://nf-co.re/isoseq) and on GitHub (https://github.com/nf-core/isoseq) under MIT License (DOI: 10.5281/zenodo.7116979).

## 1 Introduction

Genome annotation is the process of identifying transcribed and functional regions of the genome. Since the invention of high-throughput sequencing, gene, and transcript discovery has predominantly relied on the sequencing and mapping of expressed transcripts. Short-read sequencing can produce a very high number of sequences for a reasonable cost. However, as the reads are shorter than most transcripts, transcript annotation must be predicted using either *de novo* or reference-guided assembly. This step requires a lot of computation and is prone to create errors. Pacific Bioscience’s (PacBio) Iso-Seq is a long-read technology dedicated to RNA sequencing which can produce accurate full-length sequences of RNA molecules. RNA is transformed into circular double-stranded DNA, by reverse transcription, template switching, and addition of SMRTbell adapters. The molecule is sequenced continuously to produce a long sequence containing one or more copies of the original mRNA (Supplementary Fig. S1). As the copies are error prone, a multiple alignment of these copies is used to create an accurate consensus sequence of the full-length transcript. This method does not require any assembly steps, saving computation, avoiding assembly errors, and allows the direct detection of exon/intron boundaries and full-length transcript isoforms.

Numerous studies have used Iso-Seq to annotate animal (Kuo *et al.* 2017), and plant (Chao *et al.* 2019) and fish (Naftaly *et al.* 2021) genomes. Strategies vary, but all begin with the generation of Full Length Non Chimeric (FLNC) or High-Fidelity (Hi-Fi) reads using isoseq3 tools (https://github.com/PacificBiosciences/IsoSeq), followed by a mapping step and alignment post-process to compute transcript and gene models. The workflow uses several programs: ccs to compute the circular consensus sequences (CCS), lima and isoseq refine to select and clean CCS, an aligner [minimap2 (Li, 2021)], GMAP (Wu and Watanabe, 2005) and a collapsing tool such as TAMA (Kuo et al. 2020) or cupcake (https://isoseq.how).

To date, no Iso-Seq pipeline has been published. Applying this method manually by splitting data and running each program individually would be long, laborious, and prone to errors. Moreover, using FLNCs has two important advantages: (i) it avoids information compression into HiFi reads and (ii) users can run the complete workflow in parallel. Here we present nf-core/isoseq, a new nf-core pipeline (Ewels et al. 2020) for simple and hassle-free genome annotation, based on the NextFlow software. From the raw Iso-Seq subreads, it generates FLNC reads, maps them on to a reference genome, and collapses alignments to produce a genome annotation in BED format.

## 2 Pipeline description and implementation

### 2.1 Implementation

The pipeline is based on nf-core guidelines and template files. It is written using Nextflow DSL2. Each individual program in the pipeline is implemented as a module and is available as a container (Singularity, Docker, or Conda). Therefore, nf-core/isoseq requires very few dependencies to run: Java to run Nextflow (Di Tommaso et al. 2017), Nextflow to run the pipeline, and Docker or Singularity to run modules. The pipeline is composed of three parts (Supplementary Fig. S2) (i) Iso-Seq subread preprocessing; (ii) FLNC mapping; and (iii) Alignment post-processing. The input data are provided through a three-column text file listing all samples to be analysed. The columns are a unique identifier, the location of subreads in bam format, and their associated PacBio index.

### 2.2 Subread preprocessing

Iso-Seq subreads are long nucleotide sequences created from single-pass sequencing of the original mRNA molecule (Supplementary Fig. S2), available as a BAM file. As single-pass reads, they contain errors shaped by the error rate of the PacBio machine and chemistry from which they were generated. Each BAM file is processed with PacBio’s ccs program. ccs combines multiple subreads of the same molecule to produce one highly accurate consensus sequence. It can be run in parallel as it allows for splitting sequences into batches. These batches of data help to reduce execution time and are conserved until the last program (TAMA merge) of the pipeline. The generated consensus reads (“CCS reads”) still include primer sequences and polyA tails. The lima program is used to select CCS with matching primer pairs, and isoseq3 refine is used to detect and discard chimeric sequences. A final cleaning step is done by removing the remaining polyA tails using TAMA polyAcleanup helper script (Supplementary Fig. S3) after a conversion of the sequences from BAM to FASTA format with bamtools (Barnett et al. 2011).

### 2.3 Mapping

nf-core/isoseq allows the user to choose between two different aligners. uLTRA (Sahlin and Mäkinen, 2021) is a long-read aligner that outperforms other splice-aware aligners at small exon detection (<30 nucleotides). It uses the reference exon annotation to improve small exon detection and it uses minimap2 to discover unannotated genes. If no reference annotation is available for the genome, the user can map reads using minimap2.

### 2.4 Alignment post-processing

To obtain isoform annotation, each BAM file must be processed to collapse alignments (Supplementary Fig. S4). Because the data have been split at the CCS stage, collapsed annotation files must be merged by sample to create the final annotation. TAMA provides the tools for this purpose: TAMA collapse and TAMA merge. The former is used to collapse similar alignments into transcript models. The latter merges multiple annotations in one common annotation. These processes can be tuned using options to adjust identity percentage, coverage, or wobbles at 5′ end, 3′ end, and exon extremities. Resulting annotation remains unfiltered. It is up to the user to decide whether to filter the annotation, for example, based on read counts or to remove putative fused transcripts or cDNA sequences potentially primed from internal polyA sequences.

## 3 Conclusion

Iso-Seq technology can be used to detect full-length transcript isoforms. The nf-core/isoseq offers a simple solution for Iso-Seq data analysis with the execution of a series of software tools to maximize accurate extraction of information from Iso-Seq data. The produced annotation remains unfiltered, which means that no information is hidden, and users have the freedom to apply their preferred filters to improve the accuracy of the inferred transcriptome and tailor it to their study’s goals. Available online (https://nf-co.re/isoseq), the pipeline can be tested with nf-core test dataset.

## Supplementary Material

btad150_Supplementary_Data
