# Supplementary material for: nf-core/isoseq: simple gene and isoform annotation with PacBio Iso-Seq long-read sequencing
Source: Bioinformatics. 2023 Mar 24;39(5):btad150. doi: 10.1093/bioinformatics/btad150 (PMC10199315; doi:10.1093/bioinformatics/btad150)
Supplement: btad150_Supplementary_Data [file btad150_supplementary_data.pdf]

## Supplemental Data

### Supplementary Figures

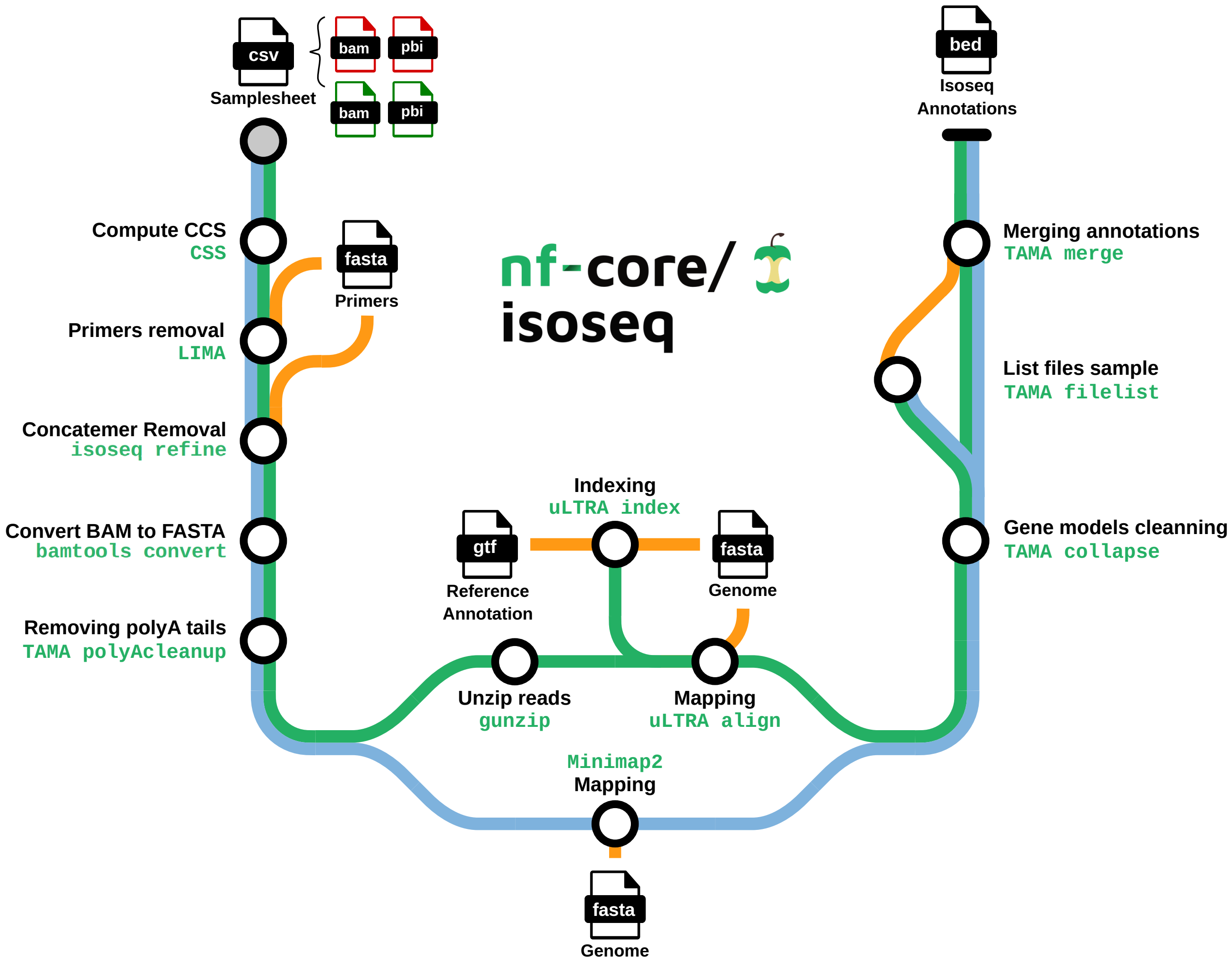


**Fig. S 1.**The nf-core/isoseq pipeline. Green and blue lines symbolize sample data. Gold line symbolizes input data.

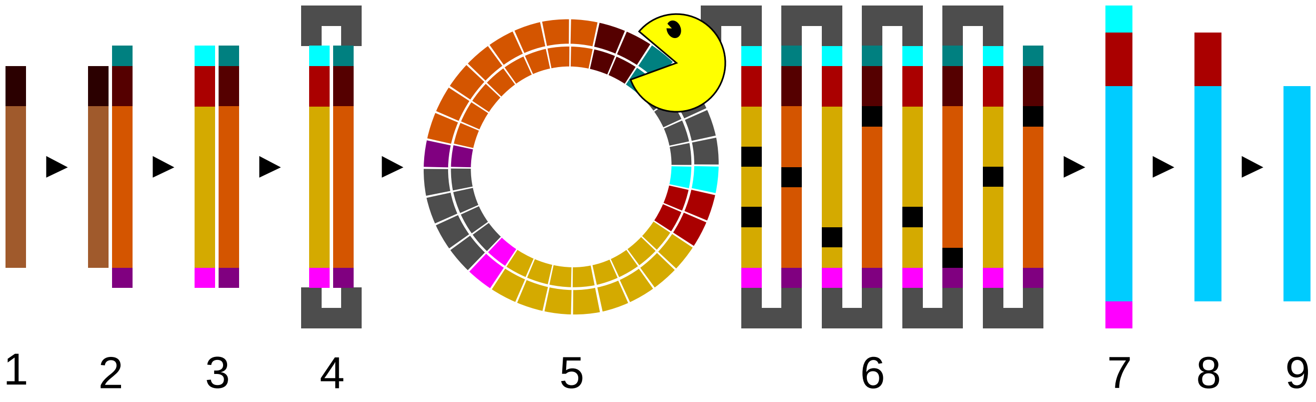


**Fig. S 2.**Iso-Seq sequencing process. 1. mRNA to sequence; 2. Reverse transcription; 3. Template switching to produce double-strand DNA; 4. Circularization and addition of SMRTbell adapters; 5. Continuous sequencing; 6. Raw subreads. Black squares symbolize sequencing errors; 7. Circular Consensus Sequence (CCS) including primers (pink and light blue squares) and polyA tail (red squares); 8. Circular Consensus Sequence (CCS) including polyA tail (red squares); 9. Full Length Non Chimeric (FLNC) reads.

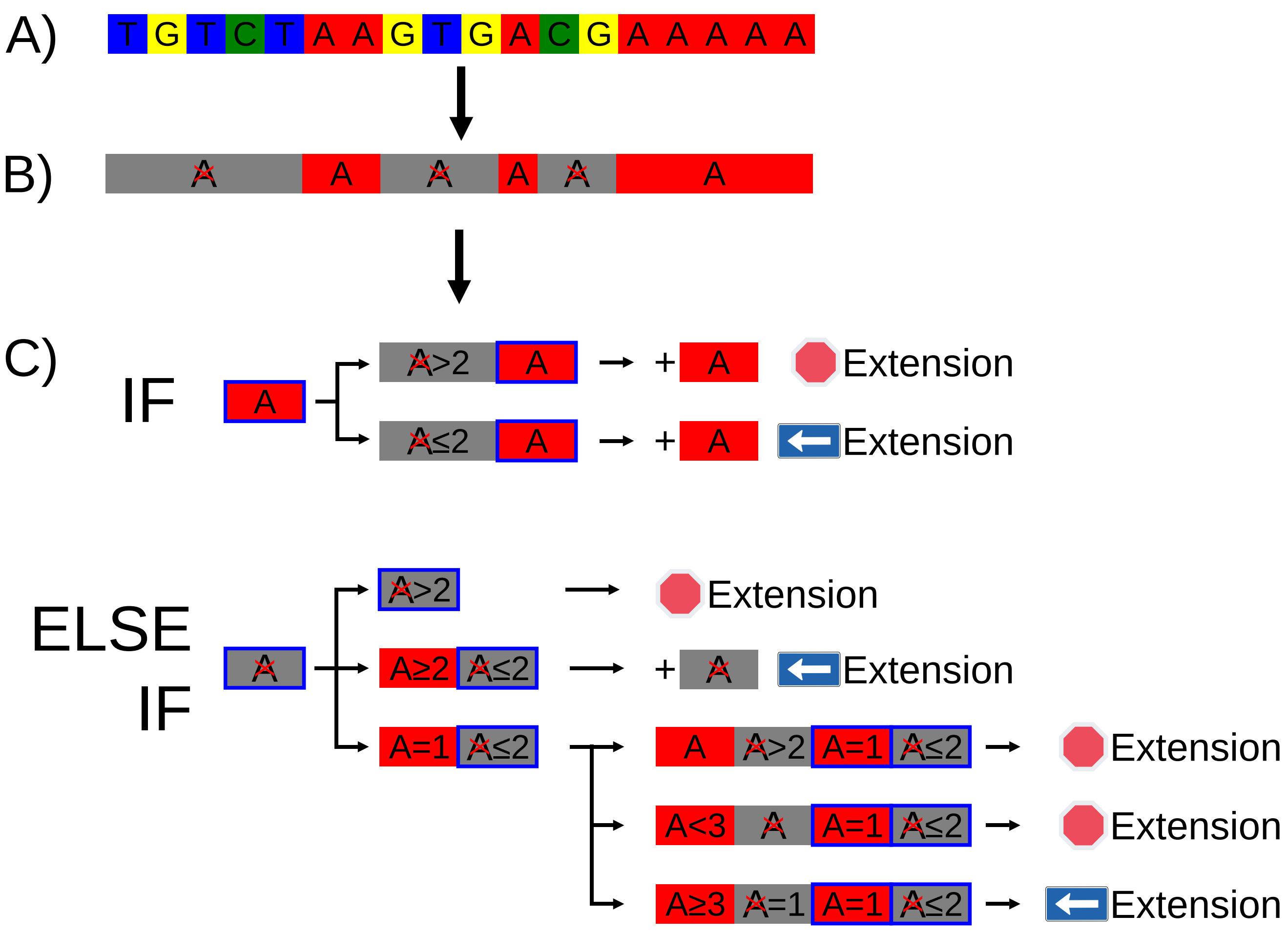


**Fig. S 3.**TAMA polyAcleanup polyA tail detection. A) FLNC with a remaining polyA tail. B) Conversion of FLNC into polyA (Red rectangles) and non polyA blocks (Grey rectangle). C) Decision tree for polyA tail detection. The decision process starts from the last blocks until extension is stopped or when third last exon is reached. Detected polyA tails are trimmed from the read.

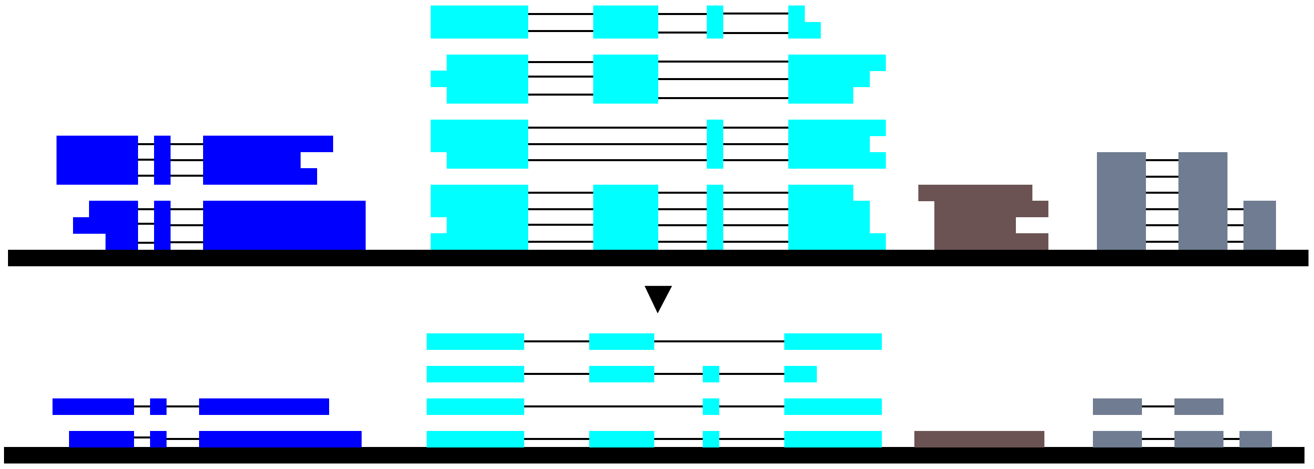

**Fig. S 4.**Transcript model creation by collapsing alignments. Black lines represent reference chromosomes. Dark blue, light blue, brown and grey lines represent reads. TAMA collapse and TAMA merge can be configured to fine tune the process. Briefly, TAMA will collapse alignments with the same chain of exons. However, using options it is possible to define wobbles at exon edges to allow flexibility in the collapsing/merging process. TAMA is also designed to take into account if libraries have been filtered on presence of 5’ cap, leading to a better annotation of 5’ end. A full description of TAMA algorithms can be found at (https://github.com/GenomeRIK/tama/wiki/)
